# The Cost of Board Examination and Preparation: An Overlooked Factor in Medical Student Debt

**DOI:** 10.7759/cureus.4168

**Published:** 2019-03-01

**Authors:** Vikrant Bhatnagar, Sebastian R Diaz, Philip A Bucur

**Affiliations:** 1 Family Medicine, Ohio University Heritage College of Osteopathic Medicine, Athens, USA; 2 Miscellaneous, Ohio University Heritage College of Osteopathic Medicine, Athens, USA

**Keywords:** medical education, board examinations, medical student debt

## Abstract

Objective

Board examinations in undergraduate medical education are imperative for competency assessment and a standard licensure process. While the cost of attendance and mean indebtedness of medical students have been quantified, the financial burden experienced by medical students from board preparation and examination has never been quantified.

Materials and methods

A total of 290 fourth-year osteopathic medical students from 38 osteopathic medical schools completed an anonymous survey that asked them to select the resources they had purchased for board preparation. Along with demographic information, respondents were asked which board examinations they had taken during their medical school education. The price for each resource was located by going to the resource website and finding the “list price” of a brand-new copy/version of that resource. If a price was not found, a current Amazon.com “list price” was utilized. These prices best approximate the maximum a student would spend per resource. Response and statistical analysis such as analysis of variance, post hoc comparison (Scheffé and Bonferronis test), and chi-square tests were conducted using the Statistical Package for Social Sciences (SPSS) Statistics, version 25.0 (IBM SPSS Statistics, Armonk, NY).

Results

This study found that osteopathic medical students spent, on average, $7,499 (s.d.=$2,506) for board preparation and examination. This cost when isolated is $3,370 for the cost of taking board examinations and $4,129 for the cost of board preparation. Respondents from the West were found to spend most at $9,432, while students from the Northeast spent the least, $7.090. Additionally, non-traditional medical students, those who matriculated after the age of 30 were found to spend more than individuals who began when they were under the age of 25 or between the ages of 25-30. The two most commonly used resources for both Level 1/Step 1 and Level 2/Step 2 examinations were COMBANK and UWorld.

Conclusions/relevance

This study is the first of its kind to quantify the mean cost of board preparation and examination in undergraduate medical education at $7,499. When considering the mean indebtedness of the osteopathic graduating class of 2017-2018, 2.94% of medical education debt can be attributed to the cost of board preparation and assessments. As competitiveness for graduate medical education increases, individuals will spend more money to ensure a competitive board exam performance, a key selection factor. Stakeholders in undergraduate medical education are encouraged to further understand the interplay between medical student debt and the cost of board examinations and preparation.

## Introduction

With every passing year, medical student debt increases [1]. A majority of medical students take out loans to pay for medical school tuition and fees, learning materials, and housing and boarding, among other expenses. For the 2017-2018 school year, the median cost of attending an osteopathic medical school was $222,972 for public schools and $261,133 for private schools [2]. Debt levels are likely to increase given that tuition costs are anticipated to rapidly increase at public medical schools [3].

Although medical student debt has been quantified, there has been scant research isolating the financial cost of board exam preparation that medical students incur during medical school. To become a licensed physician, osteopathic medical students, who comprise about a fifth of all medical students in the United States [4], must complete the Comprehensive Osteopathic Medical Licensing Examination of the United States (COMLEX-USA) Level 1, Level 2 Cognitive Evaluation (CE), Level 2 Performance Evaluation (PE), and Level 3 exams. To increase their competitiveness for residency programs, many osteopathic medical students concurrently take USMLE board examinations, the allopathic equivalent of COMLEX-USA [5]. With an additional board examination to prepare for, osteopathic medical students may opt to purchase more preparation materials. With a single accreditation system for graduate medical education almost implemented, more osteopathic medical students may take the USMLE.

The original intent of the board examination was to ensure competency assessment and a standard licensing process for future physicians. However, the results of board examinations, particularly the United States Medical Licensing Examination (USMLE) Step 1 and COMLEX Level 1, are used by residency programs to identify competitive candidates [5-6]. This has consequently heightened the focus on board examination performance. Companies including, but not limited to, Kaplan Test Preparation and Becker, have capitalized on medical students’ concerns regarding competitiveness on board examinations by offering preparation materials that they claim, if properly utilized, can lead to higher board scores [7].

Medical students also utilize external resources to augment their medical school curriculum [8]. Administrators at medical schools have noticed this trend and sometimes purchase commonly utilized resources such as first aid for the USMLE Step 1 in bulk for their students. Prior research has found that the earlier a student begins board preparation, the higher the USMLE Step 1 score [9]. Moreover, individuals who began board preparation earlier tend to have greater usage rates of first aid for the USMLE Step 1 and complete a higher number of practice questions [9].

With an increased emphasis to perform competitively on board examinations, medical students often purchase a wide array of resources. Resources come in many forms such as bundled preparation programs, review books, question banks, flashcards, videos, and other formats. The objective of this study was to quantify costs associated with board examination and preparation incurred by osteopathic medical students in the United States.

## Materials and methods

The sample size of this study comprised (*n* = 290) fourth-year osteopathic medical students from the continental United States. The Ohio University Office of Research and Sponsored Programs approved this study design and provided Institutional Review Board (IRB) approval. To reach these students, the Student Government Association (SGA) Presidents were contacted via email by the SGA President at Ohio University Heritage College of Osteopathic Medicine. Each SGA President then distributed the survey to their respective student bodies. The survey was administered over a two-week period from February 8, 2018 to February 21, 2018.

**Figure 1:**
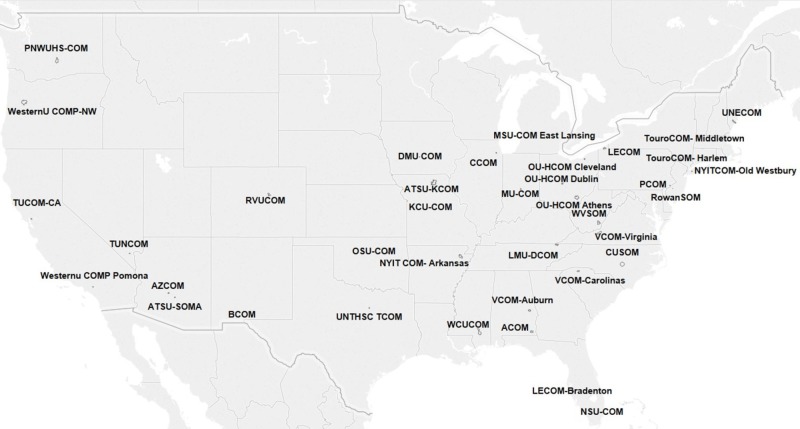
Geographic locations of the participating osteopathic medical schools

In total, 38 osteopathic medical schools and campuses chose to distribute the survey. As a result, the survey was distributed to a total of 6,162 fourth-year osteopathic medical students, out of which 290 osteopathic medical students responded, yielding a 4.71% response rate. Participating schools were located in the four main regions: Northeast (*n* = 7), West (*n* = 9), South (*n* = 12) and Midwest (*n* = 10; Figure 1).

The survey was utilized to obtain representation from the many backgrounds of osteopathic medical students and capture their different perspectives on board examination and preparation. The survey can be found in Appendix A. Qualtrics software (Provo, UT: Qualtrics Inc) was utilized to administer the survey, and the settings ensured no IP addresses were collected to guard anonymity. Furthermore, the survey did not ask for respondents’ names or medical schools. Respondents were given two weeks to participate in the study.

Participants were asked to indicate the region of the United States in which their medical school was located, their gender and race/ethnicity with which they primarily identified, and the age at which they matriculated medical school. These variables were the focus of this study as previous research has found differences in board examinations scores between races, genders, and traditional versus nontraditional students [10-12]. Participants were also asked to indicate their current year in medical school. The options for race/ethnicity were in concordance with the United States Census Bureau.

Respondents were asked about the following board examinations: 1) COMLEX - USA Level 1; 2) USMLE Step 1; 3) COMLEX-USA Level 2 CE; 4) USMLE Step 2 CK; 5) COMLEX-USA Level 2 PE, and; 6) USMLE Step 2 CS. For each exam, the respondents were given four options: 1) never taken but plan to take, 2) never taken and do not plan to take, 3) taken once, and 4) taken more than once.

Respondents were then asked to select all the listed options of resources that they had purchased in preparation for COMLEX-USA Level 1 and/or USMLE Step 1. 162 resources were provided in this comprehensive list. However, due to an oversight error, the resource First Aid for the USMLE Step 1 was not included in the list of options. An option to list “other” resources was also provided on the survey. In addition to review materials, respondents were asked how many practice exams they had purchased in preparation for USMLE Step 1 and/or COMLEX Level 1. The options included a scale from 0 to 6 for NBME Step 1 practice exams and 0 to 3 for NBOME COMSAE Phase 1 practice exams.

Participants were asked to select all the listed options of resources that they had purchased in preparation for COMLEX-USA Level 2 CE, USMLE Step 2 CK, COMLEX-USA Level 2 PE, and USMLE Step 2 CS. 135 resources were listed, and again, respondents were provided with the option of “other” to list other resources they had purchased not listed on the survey. Additionally, respondents were again asked how many practice exams they had purchased in preparation for USMLE Step 2 CK, USMLE Step 2 CS and COMLEX-USA Level 2 CE. The options included a scale from 0 to 3 for NBME Step 2 CK practice exams, 0 to 2 for NBME Step 2 CS practice exams, and 0 to 1 for NBOME Level 2 CE practice exam.

The price for each resource was located by going to the resource website and finding the “list price” of a brand-new copy/version of that resource. If a price was not found, a current Amazon.com “list price” was utilized. These prices best approximate the maximum a student would spend per resource. While an overall cost of board preparation and examination was calculated, this was separated to appreciate the individual cost of (1) board preparation and (2) examination. Responses were analyzed using the Statistical Package for Social Sciences (SPSS) Statistics, version 25.0 (IBM SPSS Statistics, Armonk, NY). 

Analysis of variance (ANOVA) was utilized to compare the means of various categories. With the nature of categorical independent variables (e.g., age of matriculation), the Scheffé test was employed to explore post hoc multiple comparisons. Otherwise, Bonferroni post hoc comparisons were utilized. Chi-square test was used to interpret categorical data. The conventional *p* = 0.05 cutoff was applied to all inferential statistical tests.

## Results

By the time osteopathic medical students graduate they spend, on average, **$7,499 **(s.d.=$2,506) for board preparation and examination.

**Figure 2:**
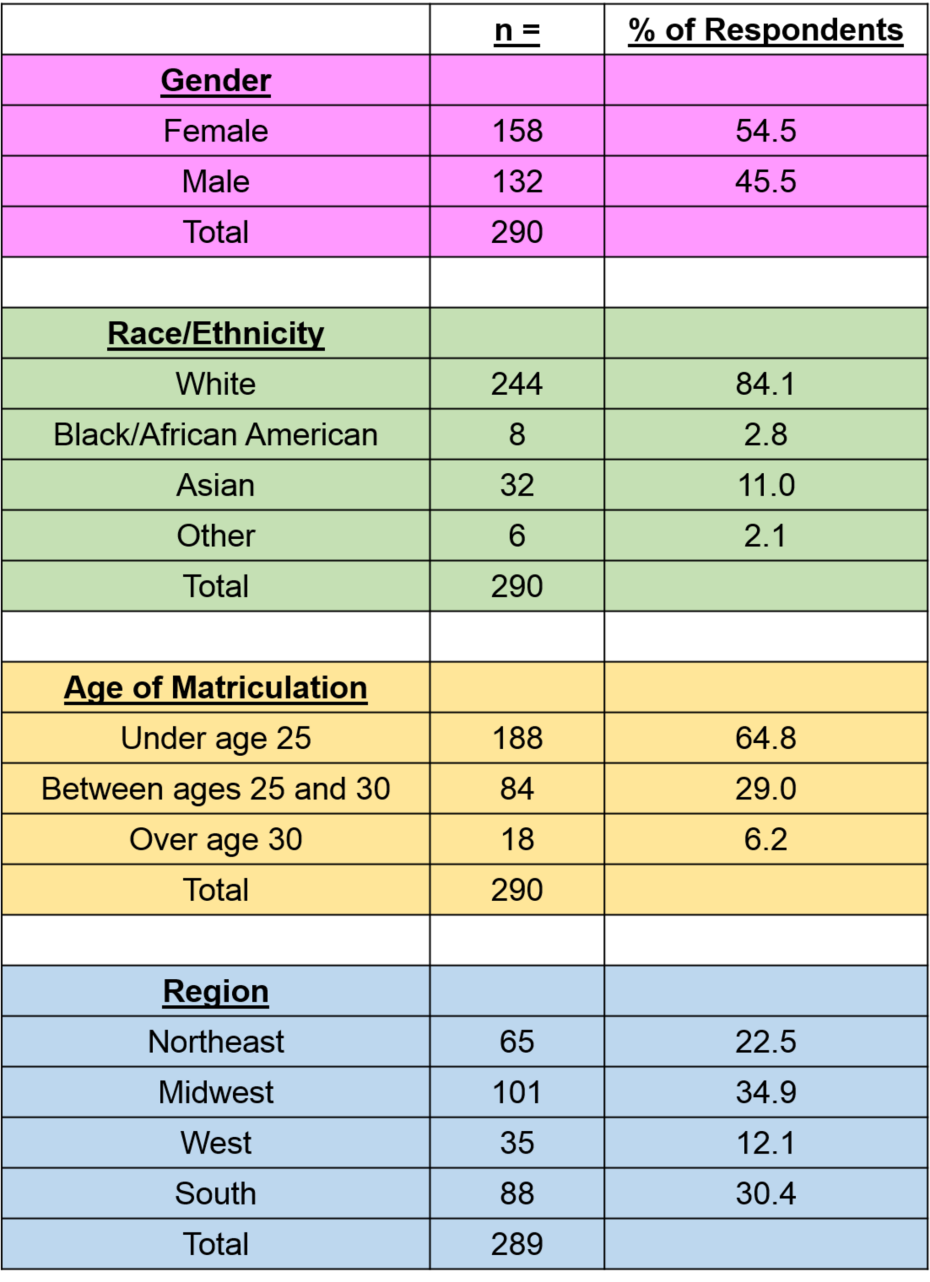
Demographic data This figure illustrates the demographic data of the survey respondents by gender, race/ethnicity, age of matriculation, and regional location of medical school. With regards to Race/Ethnicity, individuals were given multiple options to select all that applied. Due to the low sample count for American Indian or Alaska Native, and Native Hawaiian or Pacific Islander, they were aggregated as "Other".

Respondent demographics can be seen in Figure 2.

**Figure 3:**
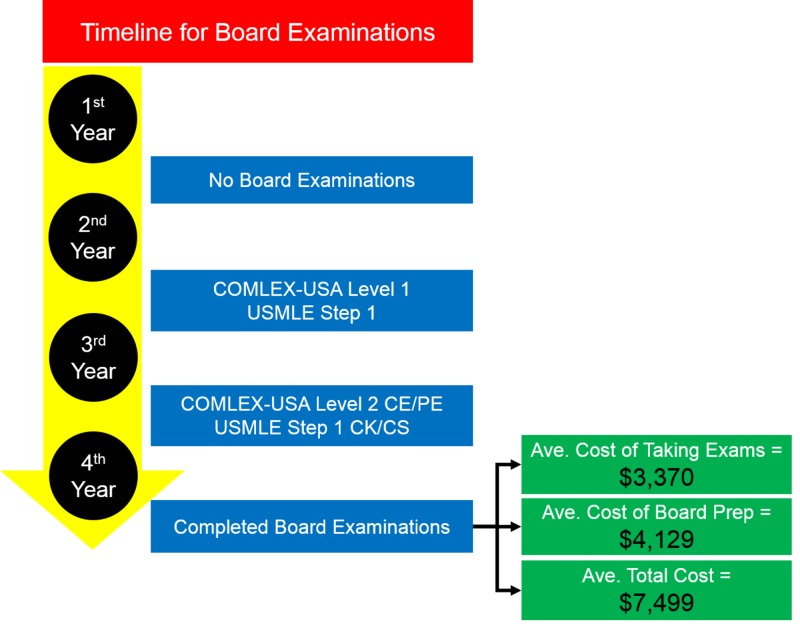
Timeline of board examinations and the various average costs incurred for taking and preparing for board examinations This illustration provides a simplistic understanding of when osteopathic medical students take board examinations and the average cost of taking examinations, average cost of board preparation, and the average total cost of both taking and preparing for board examinations.

When the total cost of both taking and preparing for board examinations is separated to 1) cost of taking board exams and 2) cost of board preparation, the impact of each cost is appreciated. The isolated cost of *taking *board examinations was $3,370 for fourth years, while the cost of board *preparation* was $4,129 for fourth years (Figure 3).

The cost of taking and preparing for board examinations did not result in much a variation when categories of race were compared. Costs accrued by Whites were $7,464 (*n *= 224), Black or African Americans spent $7,974 (*n *= 8), Asians incurred $7,674 (*n *= 32), and others accrued $7,163 (*n *= 8). ANOVA and Scheffé post hoc test did not find statistically significant differences (F = 0.34; df = 5; *p *= 0.892).

Albeit not statistically significant (F = 0.00; df = 2; *p* = 0.998), when the cost of taking and preparing for board examinations is separated by gender, males and females were found to spend nearly identical amounts with females (*n *= 158) found to have spent $7,499, while males (*n *= 132) incurred $7,498.

When respondents were categorized by the region of their medical school, respondents from the West (*n *= 35) spent the most amount equaling $9,432, while respondents from the Northeast (*n* = 65) spent the least at $7,090. Individuals from the South (*n *= 88) spent $7,379 and Midwest respondents (*n *= 101) accrued $7,199. ANOVA detected statistically significant results (F = 1.98; df = 3; *p *= 0.015). Bonferroni post hoc comparisons were detected to be statistically significant when comparing West to the other three regions (*p *< 0.001).

Categorizing respondents by age at the time of matriculation showcased an increasing trend of average cost of board examination and preparation with age. Individuals under the age 25 (*n *= 188) accrued $7,199, while respondents between ages 25-30 (*n *= 84) spent $7,721 and participants over the age 30 (*n* = 18) incurred $9,590. ANOVA detected statistically significant results (F = 8.68; df = 2; *p *< 0.001).

**Figure 4:**
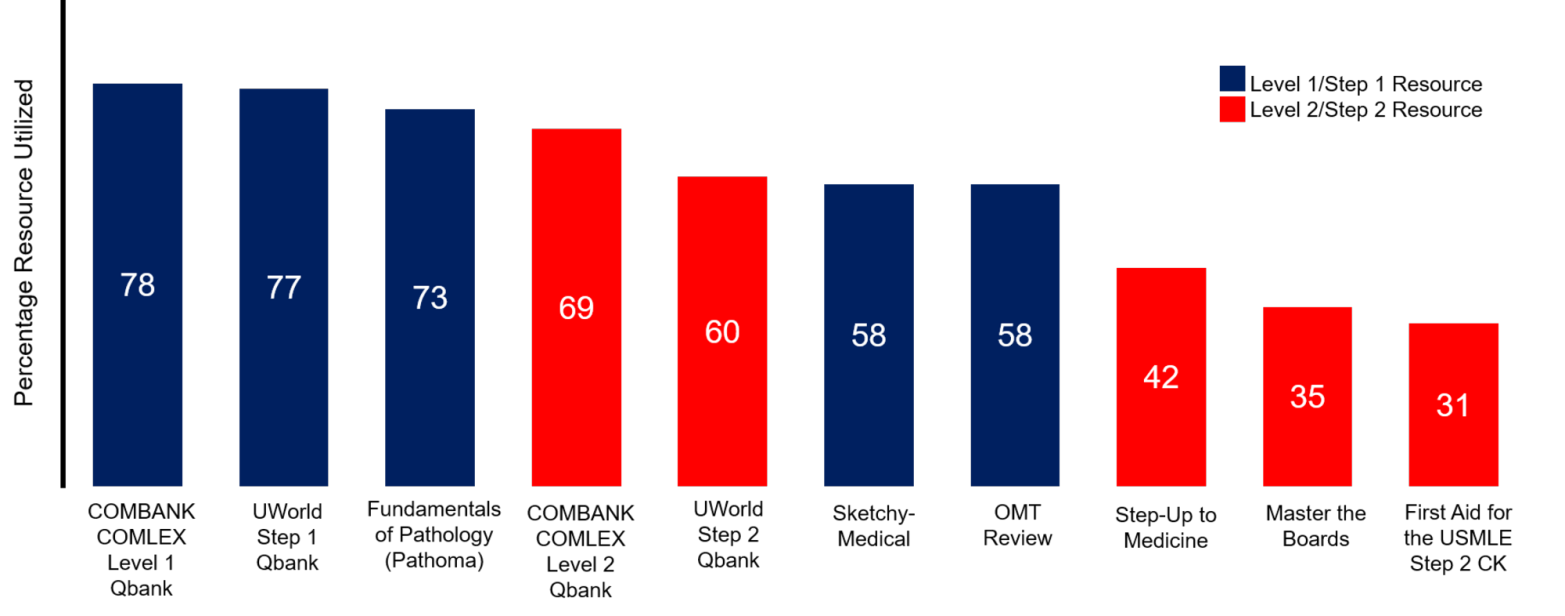
The top five resources utilized in preparation for each set of board examinations Blue highlights the resources used in COMLEX-USA Level 1 and/or USMLE Step 1, while red indicates resources utilized in COMLEX-USA Level 2 CE/PE and/or USMLE Step 2 CK/CS preparation. Percentage of resource frequency is listed in white within each column.

While the focus was to appreciate the cost of board examination and preparation, a few resources were found to be more frequently chosen by the respondents in their preparation. The five most commonly bought resources for each set of exams are depicted in Figure 4. Out of these 10 popular resources, question banks offered by COMBANK and UWorld for Level 1/Step 1 and Level 2/Step 2 were the most frequently purchased. 

## Discussion

Medical student debt, commonly calculated as the total amount of debt accrued by graduating medical students, continues to garner warranted attention in the literature. While the debt continues to rise faster than the rate of inflation, many speculate if this rate is sustainable [9,13]. This issue justifies questioning when increasing debt levels will deter potential applicants [14-15].

Although undergraduate medical education curricula are designed to fully prepare medical students for graduate medical education, students must simultaneously engage in self-directed study for board examinations. To deem a medical student a competitive applicant for a particular residency position, board performance is considered, if not the most, one of the most important qualifying factors [16]. For medical students, the major focus becomes competitive performance on board examinations, leading to substantial monies invested in board preparation material. The cost of board preparation directly affect the expenditures required to complete medical school. 

Thus far, there has been limited research on the cost of preparation for board examinations. This study quantitatively assesses the costs of taking and preparing for board examinations incurred by osteopathic medical students, which was found to be, on average, $7,499 (s.d.= $2,506) per student. The cost of board preparation and testing affects the cost of attending medical school and in this study is equivalent to 2.94% of the average student debt in the osteopathic graduating Class of 2017-2018 [2].

While the results of this study indicate that there were no statistically significant differences in the amount of money spent for board preparation and assessments between categories of race, African American or Black respondents spent approximately $500 more than White respondents. This result is in concordance with the current literature. Prior research has found that underrepresented minorities accumulate almost $30,000 more in student debt compared to White students [17]. However, it should be noted that medical student debt reflects many costs incurred by the medical student. Even so, the cost of board preparation and examination are required costs of attending medical school. These required costs aggregate and may disproportionately impact students from lower to middle financial classes, many of whom are first generation or underrepresented minorities in health care. Consequently, the high cost of medical education has been ranked highly by underrepresented minorities as the primary obstacle to pursuing a medical degree [14,18-19].

Areas for further research

Admittedly, one can speculate that osteopathic medical students spend more than allopathic medical students on board preparation since the latter are eligible for only one type of board examination (USMLE). However, a similar research study design could approximate the costs of board preparation incurred by allopathic medical students. In a similar vein, costs should also be measured for international medical graduates seeking to obtain a residency position in the United States. Together, this triumvirate of research foci can more comprehensively examine the impact of board preparation on medical student debt.

Limitations 

One limitation to note involves an oversight in the exclusion of First Aid for the USMLE Step 1 in the survey as an option. While 64 respondents identified this resource using the “Other” category, it is anticipated that other respondents who did not specify this resource had purchased it. Given a $55 list price for this resource, the figures calculated may underrepresent the total cost of board preparation by approximately 0.73%, which we believe does not significantly change the results from this study.

While 290 respondents participated in the study, the low survey response rate (4.71%) can possibly be attributed to survey fatigue by medical students, who are regularly asked to participate in surveys. Another reason that might help explain this response rate is that not only are medical students invited to participate in numerous surveys, those surveys offering monetary incentives are more likely to be completed. This survey did not financially reward students for their participation. Furthermore, some of the demographic categories that respondents were asked to identify yielded sample sizes too small to include in the study, potentially compromising the anonymity of participants.

However, this study’s respondent demographics are quite similar to the published survey data of graduating medical students from the American Association of Colleges of Osteopathic Medicine (AACOM), with their respondent demographics comprising of 44.7% female, 84.2% White, 9.0% Asian, and 3.2% Black [2,20-21]. In comparison, our respondent demographics were 54.5% female, 84.1% White, 11.0% Asian, and 2.8% Black. When comparing the age of matriculation, AACOM found 74.5% of students matriculated under the age of 25, with 19.2% between the ages of 25 and 30, and 5.4% commencing medical school after the age of 30, while our respondents were 64.8%, 29.0%, and 6.2% in the aforementioned categories [21]. Due to these similarities, we believe that the low response rate still created a sample size representative of the overall graduating osteopathic medical students, and the results found in this study can be appreciated broadly. 

Finally, other variables that may also attribute to the true cost of board preparation and examination include the total costs calculated may not be directly assumed by the respondent in all cases. At some institutions, administrators provide these resources to students at no cost. Regardless, whether directly or indirectly, medical students ultimately bear the burden of these costs.

## Conclusions

Quantifying the cost of board examination and preparation sheds light on another direct factor impacting the cost of attending medical school. Osteopathic medical students spend on average $7,499 to ensure preparedness for board examinations. With a single accreditation system almost implemented, more osteopathic medical students anticipate taking the USMLE Step 1 exam. As competitiveness for graduate medical education increases, individuals will spend more money to ensure a competitive board exam performance, a key selection factor. Stakeholders in undergraduate medical education are encouraged to further understand the interplay between medical student debt and cost of board examinations and preparation.

## Appendices

**Appendix A:** Survey: Costs Associated with Medical School Board Examination Preparation

By clicking "Yes" below, you will consent to participate in this short survey. Do you consent to participate in this study?

o Yes, I consent (I may withdraw participation at any time). (1)

o No, I do not consent. (2)

Q1 In which region of the USA is your medical school located?

o Northeast (1)

o Midwest (2)

o West (3)

o South (4)

Q2 With which of the following do you primarily identify?

o Male (1)

o Female (2)

o Other (3)

Q3 With which of the following do you primarily identify?

▢ White (1)

▢ Black or African American (2)

▢ American Indian or Alaska Native (3)

▢ Asian (4)

▢ Native Hawaiian or Pacific Islander (5)

▢ Other (6)

Q4 At what age did you BEGIN Medical School?

o Under age 25 (1)

o Between ages 25 and 30 (2)

o Over age 30 (3)

Q5 In which year of medical school are you CURRENTLY enrolled?

o OMS - I (1)

o OMS - II (2)

o OMS - III (3)

o OMS - IV (4)

o Other (e.g. dual degree, etc.) (5)

Q6 Which of the following board examinations have you taken? 

COMLEX-USA Level 1 (1)

o  Never Taken, But Plan to Take (1)

o  Never Taken, And Do NOT Plan to Take (2)

o  Taken Once (3)

o  Taken More than Once (4)

USMLE Step 1 (2)

o  Never Taken, But Plan to Take (1)

o  Never Taken, And Do NOT Plan to Take (2)

o  Taken Once (3)

o  Taken More than Once (4)

COMLEX-USA Level 2 CE (3)

o  Never Taken, But Plan to Take (1)

o  Never Taken, And Do NOT Plan to Take (2)

o  Taken Once (3)

o  Taken More than Once (4)

 USMLE  Step 2 CK (4)

o  Never Taken, But Plan to Take (1)

o  Never Taken, And Do NOT Plan to Take (2)

o  Taken Once (3)

o  Taken More than Once (4)

COMLEX-USA Level 2 PE (5)

o  Never Taken, But Plan to Take (1)

o  Never Taken, And Do NOT Plan to Take (2)

o  Taken Once (3)

o  Taken More than Once (4)

USMLE  Step 2 CS (6)

o  Never Taken, But Plan to Take (1)

o  Never Taken, And Do NOT Plan to Take (2)

o  Taken Once (3)

o  Taken More than Once (4)

Q7 Please select all resources you've purchased or plan to purchase in preparation for COMLEX-USA Level 1 / USMLE Step 1 Board Examinations.

▢ N/A (1)

▢ Acid-Base, Fluids, and Electrolytes Made Ridiculously Simple (2)

▢ Anatomy - An Essential Textbook (3)

▢ Anatomy Flash Cards: Anatomy on the Go (4)

▢ Anki (5)

▢ Atlas of Anatomy - Thieme (6)

▢ Basic Immunology (7)

▢ Becker - GuideMD (8)

▢ Becker - Integrated Cases (9)

▢ Becker - Integrated Final Review (10)

▢ Becker - Live Intensive (11)

▢ Becker - Live Online (12)

▢ Becker - Live Review (13)

▢ Becker - QMD (14)

▢ Becker - USMLE Textbooks (15)

▢ Blue Histology (16)

▢ Boards and Beyond (17)

▢ BRS Behavioral Science (18)

▢ BRS Biochemistry, Molecular Biology, and Genetics (19)

▢ BRS Cell Biology and Histology (20)

▢ BRS Embryology (21)

▢ BRS Pathology (22)

▢ BRS Pharmacology (23)

▢ BRS Physiology (24)

▢ BRS Physiology Cases and Problems (25)

▢ Case Files Anatomy (26)

▢ Case Files Biochemistry (27)

▢ Case Files Microbiology (28)

▢ Case Files Neuroscience (29)

▢ Case Files Pharmacology (30)

▢ Case Studies in Immunology: A Clinical Companion (31)

▢ Clinical Anatomy Made Ridiculously Simple (32)

▢ Clinical Biochemistry Made Ridiculously Simple (33)

▢ Clinical Biostatistics and Epidemiology Made Ridiculously Simple (34)

▢ Clinical Microbiology Made Ridiculously Simple (35)

▢ Clinical Neuroanatomy Made Ridiculously Simple (36)

▢ Color Atlas of Physiology (37)

▢ COMBANK Question Bank (38)

▢ COMQUEST Question Bank (39)

▢ Cracking the Boards: USMLE Step 1 (40)

▢ Cracking the USMLE Step 1 (41)

▢ Cram Fighter (42)

▢ Crash Course: Anatomy (43)

▢ Crash Course: Cell Biology and Genetics (44)

▢ Crash Course: Pathology (45)

▢ Crash Course: Pharmacology (46)

▢ Crush Step 1: The Ultimate USMLE Step 1 Review (47)

▢ Déjá Review: Biochemistry (48)

▢ Déjá Review: Neuroscience (49)

▢ Déjá Review: Pathology (50)

▢ Déjá Review: Pharmacology (51)

▢ Déjá Review: Physiology (52)

▢ Déjá Review: USMLE Step 1 (53)

▢ Digital Anatomist Project: Interactive Atlases (54)

▢ Doctors in Training COMLEX Level 1 Bundle (55)

▢ Doctors in Training Gross Anatomy & Radiology Study Guide (56)

▢ Doctors in Training OMM Review (57)

▢ Doctors in Training USMLE Step 1 Review Course (58)

▢ Dr. Najeeb Lectures (59)

▢ Elsevier's Integrated Immunology and Microbiology (60)

▢ Elsevier's Integrated Pharmacology (61)

▢ Elsevier's Integrated Physiology (62)

▢ Elsevier's Integrated Review: Genetics (63)

▢ Endocrine Physiology (64)

▢ Firecracker (65)

▢ First Aid Cases for the USMLE Step 1 (66)

▢ First Aid for the Basic Sciences: General Principles (67)

▢ First Aid for the Basic Sciences: Organ Systems (68)

▢ First Aid Q&A for the USMLE Step 1 (69)

▢ First Aid Step 1 Express (70)

▢ First Aid Step 1 Flash Facts (71)

▢ Goljan Rapid Review Pathology (72)

▢ Gray's Anatomy for Students Flash Cards (73)

▢ Hematology at a Glance (74)

▢ High-Yield Behavioral Science (75)

▢ High-Yield Biostatistics, Epidemiology, and Public Health (76)

▢ High-Yield Cell and Molecular Biology (77)

▢ High-Yield Embryology (78)

▢ High-Yield Gross Anatomy (79)

▢ High-Yield Histopathology (80)

▢ High-Yield Neuroanatomy (81)

▢ Jekel's Epidemiology, Biostatistics, Preventive Medicine, and Public Health (82)

▢ Kaplan Level 1 Qbank (83)

▢ Kaplan Medical Anatomy Flash Cards (84)

▢ Kaplan Medical USMLE Medical Ethics (85)

▢ Kaplan USMLE Step 1 Prep - In Center (86)

▢ Kaplan USMLE Step 1 Prep - Live (87)

▢ Kaplan USMLE Step 1 Prep - Live Online (88)

▢ Kaplan USMLE Step 1 Prep - On Demand (89)

▢ Kaplan USMLE Step 1 Qbank (90)

▢ Kaplan USMLE Step 1 Qbook (91)

▢ Katzung & Trevor's Pharmacology: Examination and Board Review (92)

▢ Lange Biochemistry and Genetics Flash Cards (93)

▢ Lange Microbiology & Infectious Diseases Flash Cards (94)

▢ Lange Pathology Flash Cards (95)

▢ Lange Pharmacology Flash Cards (96)

▢ Lippencott's Microcards: Microbiology Flash Cards (97)

▢ Lippincott's Illustrated Q&A Review of Rubin's Pathology (98)

▢ Lippincott's Illustrated Reviews: Biochemistry (99)

▢ Lippincott's Illustrated Reviews: Immunology (100)

▢ Lippincott's Illustrated Reviews: Microbiology (101)

▢ Lippincott's Illustrated Reviews: Pharmacology (102)

▢ Master the Boards USMLE Step 1 Pharmacology Flash Cards (103)

▢ medEssentials for the USMLE Step 1 (104)

▢ Medical Biochemistry - An Illustrated Review (105)

▢ Medical Microbiology and Immunology Flash Cards (106)

▢ Medical School Pathology (107)

▢ Memorang (108)

▢ Netter's Anatomy Flash Cards (109)

▢ Netter's Physiology Flash Cards (110)

▢ Northwestern Medical Review - Live Review Pack (111)

▢ Northwestern Medical Review - Online COMLEX Level 1 Review (112)

▢ Northwestern Medical Review - Online USMLE Step 1 Review (113)

▢ OMT Review: A Comprehensive Review in Osteopathic Medicine by Savarese (114)

▢ Osmosis (115)

▢ Packet Companion to Robbins and Cotran Pathologic Basis of Disease (116)

▢ Pass Program COMLEX-USA Live On-Site Program (117)

▢ Pass Program USMLE Step 1 Live Online Program (118)

▢ Pass Program USMLE Step 1 Live On-Site Program (119)

▢ Pathoma: Fundamentals of Pathology (120)

▢ Pathophysiology of Disease: Introduction to Clinical Medicine (121)

▢ PharmCards: Review Cards for Medical Students (122)

▢ Physeo (123)

▢ Picmonic (124)

▢ PreTest: Biochemistry and Genetics (125)

▢ PreTest: Clinical Vignettes for the USMLE Step 1 (126)

▢ Pretest: Microbiology (127)

▢ PreTest: Neuroscience (128)

▢ PreTest: Pathology (129)

▢ PreTest: Pharmacology (130)

▢ PreTest: Physiology (131)

▢ Pulmonary Pathophysiology: The Essentials (132)

▢ Radiopaedia (133)

▢ Rapid Review: Biochemistry (134)

▢ Rapid Review: Gross and Developmental Anatomy (135)

▢ Rapid Review: Microbiology and Immunology (136)

▢ Rapid Review: Pathology (137)

▢ Rapid Review: Pharmacology (138)

▢ Rapid Review: Physiology (139)

▢ Review of Medical Microbiology and Immunology (140)

▢ Review of Microbiology & Immunology (141)

▢ Robbins and Cotran Review of Pathology (142)

▢ SketchyMedical (143)

▢ Step-Up to USMLE Step 1 (144)

▢ The Pathology Guy (145)

▢ The Whole Brain Atlas (146)

▢ USMLE Consult (147)

▢ USMLE Images for the Boards: A Comprehensive Image-Based Review (148)

▢ USMLE Step 1 Made Ridiculously Simple (149)

▢ USMLE Step 1 Secrets in Color (150)

▢ USMLE Success Academy Live Step 1 Prep Program (151)

▢ USMLE Success Academy Online + Live Step 1 Prep Program (152)

▢ USMLE Success Academy Online Step 1 Prep Program (153)

▢ USMLEagle Prep - Step 1 (154)

▢ USMLE-Rx Step 1 Qmax (155)

▢ UWorld Qbank (156)

▢ Vander's Renal Physiology (157)

▢ WebPath: The Internet Pathology Laboratory (158)

▢ Wheater's Functional Histology: A Text and Colour Atlas (159)

▢ WolfPacc COMLEX Level 1 (160)

▢ WolfPacc USMLE Step 1 (161)

▢ Other (162) ________________________________________________

Q8 How many PRACTICE EXAMS did you purchase or plan to purchase in preparation for COMLEX-USA Level 1 / USMLE Step 1 Board Examinations?

NBME Step 1 (1)

o 0 (1)

o 1 (2)

o 2 (3)

o 3 (4)

o 4 (5)

o 5 (6)

o 6 (7)

NBOME Step 1 (2)

o 0 (1)

o 1 (2)

o 2 (3)

o 3 (4)

o (5)

o (6)

o (7)

Q9 Please select all resources you’ve purchased or plan to purchase in preparation for COMLEX-USA Level 2 CE / USMLE Step 2 CK Board Examinations

▢ N/A (1)

▢ A & L’s Review of Psychiatry (2)

▢ A & L’s Review of Surgery (3)

▢ Abernathy’s Surgical Secrets (4)

▢ Blueprints Clinical Cases in Family Medicine (5)

▢ Blueprints Clinical Cases in Medicine (6)

▢ Blueprints Clinical Cases in Neurology (7)

▢ Blueprints Clinical Cases in Obstetrics and Gynecology (8)

▢ Blueprints Clinical Cases in Pediatrics (9)

▢ Blueprints Clinical Cases in Psychiatry (10)

▢ Blueprints Clinical Cases in Surgery (11)

▢ Blueprints Medicine (12)

▢ Blueprints Neurology (13)

▢ Blueprints Obstetrics and Gynecology (14)

▢ Blueprints Pediatrics (15)

▢ Blueprints Psychiatry (16)

▢ Blueprints Q & A Step 2 Obstetrics & Gynecology (17)

▢ Blueprints Q & A Step 2 Pediatrics (18)

▢ Blueprints Q & A Step 2 Psychiatry (19)

▢ Blueprints Surgery (20)

▢ Boards & Wards for USMLE Step 2 (21)

▢ Case Files Emergency Medicine (22)

▢ Case Files Family Medicine (23)

▢ Case Files Internal Medicine (24)

▢ Case Files Obstetrics and Gynecology (25)

▢ Case Files Pediatrics (26)

▢ Case Files Psychiatry (27)

▢ Case Files Surgery (28)

▢ Clinical Vignettes for the USMLE Step 2 CK: PreTest Self-Assessment & Review (29)

▢ COMBANK Question Bank (30)

▢ COMQUEST Question Bank (31)

▢ Crush Step 2: The Ultimate USMLE Step 2 Review (32)

▢ Déjà Review: Emergency Medicine (33)

▢ Déjà Review: Family Medicine (34)

▢ Déjà Review: Internal Medicine (35)

▢ Déjà Review: Obstetrics and Gynecology (36)

▢ Déjà Review: Pediatrics (37)

▢ Déjà Review: Psychiatry (38)

▢ Déjà Review: Surgery (39)

▢ Déjà Review: USMLE Step 2 CK (40)

▢ Doctors in Training COMLEX Level 2 Bundle (41)

▢ Doctors in Training USMLE Step 2 CK Review Course (42)

▢ Dr. Pestana's Surgery Notes: Top 180 Vignettes for the Surgical Wards (43)

▢ Emergency Medicine: PreTest Self-Assessment and Review (44)

▢ Family Medicine: PreTest Self-Assessment and Review (45)

▢ First Aid Cases for the USMLE Step 2 CK (46)

▢ First Aid for the Emergency Medicine Clerkship (47)

▢ First Aid for the Medicine Clerkship (48)

▢ First Aid for the Obstetrics & Gynecology Clerkship (49)

▢ First Aid for the Pediatrics Clerkship (50)

▢ First Aid for the Psychiatry Clerkship (51)

▢ First Aid for the Surgery Clerkship (52)

▢ First Aid for the Wards (53)

▢ First Aid for USMLE Step 2 CK (54)

▢ First Aid Q&A for the USMLE Step 2 CK (55)

▢ High-Yield Internal Medicine (56)

▢ High-Yield Obstetrics and Gynecology (57)

▢ High-Yield Psychiatry (58)

▢ High-Yield Surgery (59)

▢ Images from the Wards: Diagnosis and Treatment (60)

▢ In A Page Emergency Medicine (61)

▢ In A Page Pediatrics (62)

▢ In A Page Surgery (63)

▢ Kaplan USMLE Step 2 CK Prep - In Center (64)

▢ Kaplan USMLE Step 2 CK Prep - Live (65)

▢ Kaplan USMLE Step 2 CK Prep - Live Online (66)

▢ Kaplan USMLE Step 2 CK Prep - On Demand (67)

▢ Kaplan USMLE Step 2 QBank (68)

▢ Lange Outline Review: USMLE Step 2 (69)

▢ Lange Practice Tests: USMLE Step 2 (70)

▢ Lange Q&A: Internal Medicine (71)

▢ Lange Q&A: Obstetrics and Gynecology (72)

▢ Lange Q&A: Pediatrics (73)

▢ Lange Q&A: Psychiatry (74)

▢ Lange Q&A: Surgery (75)

▢ Lange Q&A: USMLE Step 2 (76)

▢ Master the Boards USMLE Step 2 CK (77)

▢ Medicine: PreTest Self-Assessment & Review (78)

▢ Neurology Recall (79)

▢ Neurology Secrets (80)

▢ Neurology: PreTest Self-Assessment & Review (81)

▢ NMS Obstetrics and Gynecology (82)

▢ NMS Pediatrics (83)

▢ NMS Psychiatry (84)

▢ NMS Review for USMLE Step 2 CK (85)

▢ NMS Surgery (86)

▢ Northwestern Medical Review - Online COMLEX Level 2 CE Review (87)

▢ Northwestern Medical Review - Online USMLE Step 2CK Review (88)

▢ Obstetrics and Gynecology Recall (89)

▢ Obstetrics and Gynecology Secrets (90)

▢ Obstetrics and Gynecology: PreTest Assessment & Review (91)

▢ Pass Program USMLE Step 2 Live On-Demand Program (92)

▢ Pass Program USMLE Step 2 Live Online Program (93)

▢ Pass Program USMLE Step 2 Live On-Site Program (94)

▢ Pediatric Secrets (95)

▢ Pediatrics Recall (96)

▢ Pediatrics: PreTest Self-Assessment & Review (97)

▢ Physical Diagnosis: PreTest Self-Assessment & Revie (98)

▢ Psychiatry: PreTest Self-Assessment & Review (99)

▢ Review 2 Rounds: Visual Review and Clinical Reference (100)

▢ Step-Up to Medicine (101)

▢ Step-Up to USMLE Step 2 CK (102)

▢ Surgery: PreTest Self-Assessment & Review (103)

▢ Surgical Recall (104)

▢ Underground Clinical Vignettes Step 2: Emergency Medicine (105)

▢ Underground Clinical Vignettes Step 2: Internal Medicine Vols. I and II (106)

▢ Underground Clinical Vignettes Step 2: Neurology (107)

▢ Underground Clinical Vignettes Step 2: OB/GYN (108)

▢ Underground Clinical Vignettes Step 2: Pediatrics (109)

▢ Underground Clinical Vignettes Step 2: Psychiatry (110)

▢ Underground Clinical Vignettes Step 2: Surgery (111)

▢ Underground Clinical Vignettes: Step 2 Bundle (112)

▢ USMLE Consult’s Step 2 CK Question Bank (113)

▢ USMLE Road Map: Emergency Medicine (114)

▢ USMLE Step 2 Mock Exam (115)

▢ USMLE Step 2 Recall (116)

▢ USMLE Step 2 Secrets (117)

▢ USMLE Success Academy Live Step 2 CK Prep Program (118)

▢ USMLEagle Prep - Step 2 (119)

▢ USMLEasy (120)

▢ USMLE-Rx USMLE Step 2 Qmax (121)

▢ Uworld Step 2 CK Qbank (122)

▢ WolfPacc COMLEX Level 1 (123)

▢ WolfPacc USMLE Step 2 CK (124)

▢ Other (125) ________________________________________________

Q10 Please select all resources you’ve purchased or plan to purchase in preparation for COMLEX-USA Level 2 PE / USMLE Step 2 CS Board Examinations?

▢ N/A (1)

▢ Blueprints USMLE Step 2 CS (2)

▢ CS Checklists: Portable Review for the USMLE Step 2 CS (3)

▢ First Aid for the USMLE Step 2 CS (4)

▢ Mastering the USMLE Step 2 CS (5)

▢ NMS Review for the USMLE Clinical Skills Exam (6)

▢ Pass Program USMLE Step 2 Clinical Skills Program (7)

▢ The Ultimate Guide and Review for the USMLE Step 2 Clinical Skills Exam (8)

▢ USMLE Step 2 CS: Complex Cases-35 Cases You Are Likely to See on the Exam (9)

▢ Other (10) ________________________________________________

Q11 How many PRACTICE EXAMS did you purchase or plan to purchase in preparation for USMLE Step 2 CK / USMLE Step 2 CS / COMLEX-USA Level 2 CE?

NBME Step 2 CK (1)

o 0 (1)

o 1 (2)

o 2 (3)

o 3 (4)

NBME Step 2 CS (2)

o 0 (1)

o 1 (2)

o 2 (3)

o (4)

NBOME Level 2 CE (3)

o 0 (1)

o 1 (2)

o (3)

o (4)

Q12 Please evaluate the following: 

I plan to enter a Primary Care Residency (Family Medicine OR General Internal Medicine OR Pediatrics) (1)

o  Strongly Disagree (1)

o  Disagree (2)

o  Neutral (3)

o  Agree (4)

o  Strongly agree (5)

Preparation for Board Examinations influenced how I approached medical school. (2)

o  Strongly Disagree (1)

o  Disagree (2)

o  Neutral (3)

o  Agree (4)

o  Strongly agree (5)

I would advise future medical students to carefully plan for the cost of preparation for Board Examinations. (3)

o  Strongly Disagree (1)

o  Disagree (2)

o  Neutral (3)

o  Agree (4)

o  Strongly agree (5)

Studying for medical school courses took priority over studying for Board Examinations. (4)

o  Strongly Disagree (1)

o  Disagree (2)

o  Neutral (3)

o  Agree (4)

o  Strongly agree (5)

 My medical school's curriculum prepared me for Board Examinations. (5)

o  Strongly Disagree (1)

o  Disagree (2)

o  Neutral (3)

o  Agree (4)

o  Strongly agree (5)

My school purchased Board Examination preparation material that was integral for my preparation. (6)

o  Strongly Disagree (1)

o  Disagree (2)

o  Neutral (3)

o  Agree (4)

o  Strongly agree (5)
